# Physiotherapy Screening for Referral of a Patient with Peripheral Arterial Disease Masquerading as Sciatica: A Case Report

**DOI:** 10.3390/healthcare11111527

**Published:** 2023-05-24

**Authors:** Daniel Feller, Andrea Giudice, Giorgio Maritati, Filippo Maselli, Giacomo Rossettini, Roberto Meroni, Graziana Lullo, Nathan Hutting, Firas Mourad

**Affiliations:** 1 Provincial Agency for Health of the Autonomous Province of Trento, Centre of Higher Education for Health Sciences, 38122 Trento, Italy; 2 Department of Physiotherapy, Poliambulatorio Physio Power, 25124 Brescia, Italy; 3 Department of Human Neurosciences, Sapienza University of Rome, 00185 Rome, Italy; 4 Department of Physiotherapy, LUNEX International University of Health, Exercise and Sports, 4671 Differdange, Luxembourg; 5 Luxembourg Health and Sport Sciences Research Institute A.s.b.l., 50, Avenue du Parc des Sports, 4671 Differdange, Luxembourg; 6 Department of Physiotherapy, Fisiopiù Poliambulatorio, 70038 Bari, Italy; 7 Department of Occupation and Health, School of Organisation and Development, HAN University of Applied Sciences, 6503 GL Nijmegen, The Netherlands

**Keywords:** differential diagnosis, ankle brachial index, claudication, sciatica, rehabilitation

## Abstract

Many causes potentially underline pain in the lower extremities, presenting a real challenge for primary care clinicians in the recognition of the source of the patient’s complaints. Peripheral arterial disease (PAD) is defined as a total or partial blockage of the vessels that supply blood from the heart to the periphery. PAD of the lower extremities may masquerade as lumbosacral radiculopathy (LSR)—a common source of leg pain. Physiotherapists should be able to screen for PAD in people presenting with pain in the lower extremities. Failure to correctly screen for PAD could put the patient at risk of severe disability and possible permanent sequelae. This case report outlines the relevant concepts relating to the pathophysiology, screening, and differential diagnosis of PAD, and then further describes the relevant findings from the history and physical examination from the physiotherapist’s perspective in a patient with an unusual symptom presentation. Although the patient was referred by a physician with a diagnosis of LSR, our case highlights the pivotal role of skilled physiotherapists in triaging a severe lower-limb PAD in need of referral. Therefore, this case report aims to increase clinicians’ awareness of the clinical features of a complex case of PAD.

## 1. Introduction

Many causes potentially underline pain in the lower extremities, presenting a real challenge for primary care clinicians in the recognition of the source of the patient’s complaints [1,2,3,4,5]. One of the most common sources of leg pain is lumbosacral radiculopathy (LSR)—a neuroinflammatory process that impairs one or more lumbosacral nerve roots [6]. Between 2 and 5 percent of the general population suffer from LSR, and most of the time the first-line treatment is conservative (e.g., physiotherapy) [6]. Neurological deficit (namely, loss of function) is a core feature of LSR, resulting in muscle weakness, reduced deep tendon reflexes, and sensational changes [7]. LSR also often presents neuropathic pain (namely, gain of function), reported as pain radiating down to one or both legs [7]. In a recent modified Delphi study, the interviewed experts agreed that LSR’s pain is mainly characterized by the quality of the symptoms, which the patient describes as burning, electric shock and/or shooting into the leg, allodynia and/or hyperpathia within the distribution of the pain, spontaneous pain and/or paroxysmal pain, and pain described as crawling or other unpleasant abnormal sensations [8]. Although LSR’s pain features are well defined, it is often challenging to clinically identify patients with neuropathic pain in lower-back-related leg pain [8].

Another common potential cause of pain in the lower extremities is peripheral arterial disease (PAD) [9]. PAD is defined as a total or partial blockage of the vessels that supply blood from the heart to the periphery [10]. The prevalence of PAD is around 7% in subjects aged between 55 and 59 years, reaching almost 25% in individuals between 95 and 99 years old [11]. Known risk factors for PAD include diabetes, hypertension, dyslipidemia, hyperhomocysteinemia, C-reactive protein levels, renal insufficiency, and smoking. When symptomatic, PAD is characterized by lower-limb pain, which can masquerade as LSR in the early stages [1]. Progressively, people with PAD may present reduced skin temperature with discoloration, air loss, hypertrophic and ridged nails and, in extreme cases, focal areas of ischemia with full-thickness skin necrosis [12,13].

The population around the world is aging, with increased medical complexity and morbidities; therefore, physiotherapists must be capable of triaging PAD to determine whether primary care referral is required for optimal management [1,14].

This case report highlights the importance of screening for PAD in those patients presenting with pain in the lower extremities in a direct-access physiotherapy setting [15,16,17]. Recognition of red flags associated with PAD in the hypothesis generation phase requires knowledge of pathophysiology, clinical presentation, and diagnostic testing [18,19,20]. Failure to correctly screen for (i.e., the physiotherapist) or diagnose (i.e., the medical physician) PAD could place the patient at risk of severe disability and possible permanent sequelae [21]. Therefore, our case report aims to increase clinicians’ awareness of the clinical features of a complex case of PAD. That is, the patient in the present case was referred by a physician with a diagnosis of LSR for physiotherapy treatment. He was also recently examined by a vascular specialist, who ruled out a vascular origin of his symptoms. However, the knowledge, a sound clinical reasoning, and a proper subjective and clinical examination increased the suspicion of severe lower-limb PAD in need of referral. We recommend that readers integrate this case with the Professional Issue of Feller et al. [1], which provides relevant aspects of the clinical reasoning process and the essentials of the peripheral cardiovascular objective examination. This Professional Issue highlights the main risk factors and symptoms to investigate during the patient interview, the clinical reasoning, and the role of physiotherapy in PAD management. Moreover, it provides a step-by-step guide on how to objectively assess a patient suspected of having PAD, including pulse palpation, measurements of vital signs, and the application and interpretation of the ankle-brachial index.

This case report follows the CARE checklist [22].

## 2. Case Presentation

### 2.1. Patient Information

A 67-year-old male attended our physiotherapy clinic after being referred by a neurosurgeon for bilateral sciatica—a term commonly used to describe lower-back-related leg pain. Four years prior to the physiotherapy consultation, the patient had a stroke at the left medulla oblongata that caused a Wallenberg syndrome associated with temperature and pain sensation deficits on the right side, associated with vertigo and nystagmus. Since this episode, he had been under pharmacological treatment, taking aspirin, blood-pressure medication, cholesterol, and antidiabetic drugs. One year later, he underwent a coronary bypass because of a severe occlusion of two coronary arteries, diagnosed following an angina episode, and then an aortic aneurysm surgical repair. He was diabetic and a smoker with a sedentary lifestyle; although diabetic, his diet was unbalanced, with a high red meat intake and high alcohol consumption.

His right sciatica symptoms started one year prior to the physiotherapy consultation, with a primary complaint of knee pain associated with lower-back pain; his right-knee pain quickly developed into burning pain on the right (mainly posteriorly) and progressed to the anterior tibia and the toe. In the following months, the symptoms fluctuated in location and intensity, leading the patient to an orthopedic consultation. The following knee magnetic resonance imaging (MRI) and the thigh ultrasound did not reveal any local abnormalities. However, he started having posterior left-thigh–knee pain over time, and the patient visited a physiatrist and neurosurgeon for consultation. Based on a lower-back MRI scan and computed tomography scans, which revealed an L4-5 and L5-S1 wide-disk bulging with an L5 root impingement, the neurosurgeon diagnosed bilateral LSR. The referring neurosurgeon treated him with epidural corticosteroids, anesthetic injection, and ozone therapy, achieving a reduction in the left-leg and lower-back symptoms, but without any benefits for the right leg.

**Figure 1:**
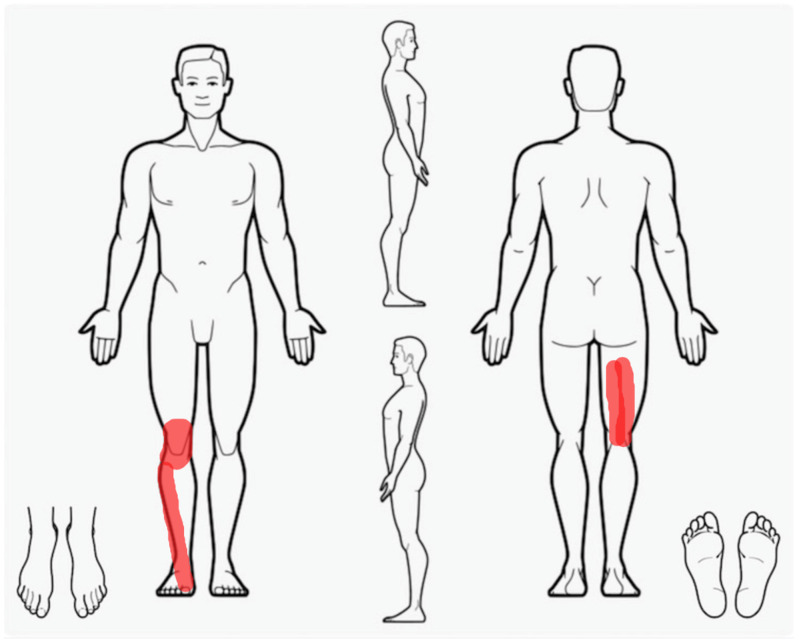
The right lower extremity symptoms’ localization at the time of the first clinical encounter.

**Figure 2:**
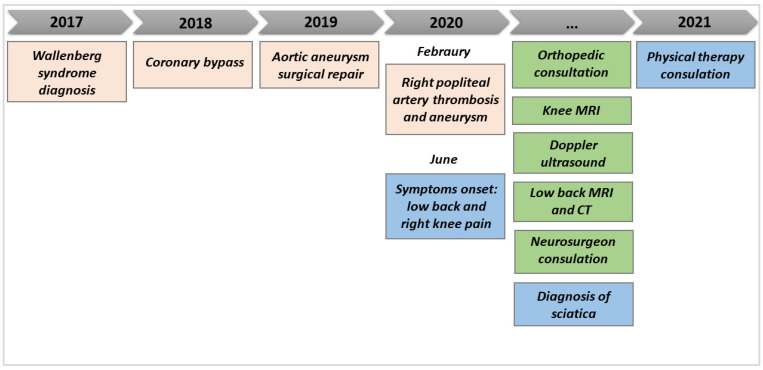
Medical history timeline prior to the physiotherapy consultation.

Furthermore, as he had recently had a right popliteal artery thrombosis and aneurysm surgically treated with an endoprosthesis, a Doppler ultrasound was prescribed, which still showed the thrombosis of the popliteal artery but also revealed a good vascular compensation of the anterior lower limb due to the recanalization of the origin of the anterior and the middle portion of the posterior tibial artery. After the objective examination—no night pain and only slight hypothermia—the vascular surgeon did not advise any supplementary revascularization surgery, but anticoagulant and antiaggregant pharmacological therapies were prescribed in addition to his usual medication intake. At the physiotherapy consultation, the resting pain was 6/10 on the numeric pain rating scale. The symptoms were located anteriorly and posteriorly on the right up to the foot and were worsened by activities of daily living, such as walking, limiting him to 1000 steps per day (Figure 1). For further details on the patient’s medical history, refer to the timeline in Figure 2.

### 2.2. Patient Assessment

A neurological examination was performed to evaluate the function of the peripheral nervous system. Although the interpretation of the findings was difficult because of the Wallenberg syndrome, pain and thermal sensibility were reduced on the right side. Muscle force and deep tendon reflexes were unremarkable and comparable between sides. SLUMP and SLR testing [23] reproduced his previous left-thigh–knee pain; when sustained on the right side, his lower-limb symptoms increased (i.e., pain and paresthesia). There was an observable muscle spasm on the erector spinae bilaterally, and lumbar active flexion and extension were limited and painful. Skin texture changes—including thin and brittle skin—and pallor on the right tibia and foot, along with decreased skin temperature of the right foot, were observed upon visual observation and inspection. Therefore, no further conventional testing (e.g., ROM and strength) was performed.

Although assessed by his vascular physician, based on the impossibility of performing a proper neurological examination and the recent unusual presentation (i.e., incurable high-intensity pain, burning and tingling-like pain quality on the whole lower leg, with unpredictable localization), the skin texture changes, and the presence of cardiovascular risk factors (i.e., popliteal artery thrombosis, abdominal aortic aneurysm, lifestyle, diet, comorbidities such as hyperglycemia and high- cholesterol levels), measurements of vital signs, blood pressure (BP), and vascular testing were performed [1]. The temperature was average; the pulse was 55 bpm and regular; the mean BP of three measurements taken on the left was 120/80 mm Hg. The popliteal and posterior tibial BP was also taken, showing normal results for both measurements on the left, but undetectable on the right leg. During manual palpation, the popliteal and posterior tibial pulse was weak and almost undetectable. Sustained compression in the inguinal canal during femoral artery pulse palpation reproduced the tingling pain on the anterior thigh; auscultation at the femoral triangle and the popliteal fossa was difficult and did not reveal any apparent bruit. No pulsating mass was palpable during palpation of the lower-limb arteries. During the plantar flexion test [1], the patient’s familiar knee pain was reproduced after 10 repetitions and progressively spread over the lower limb as burning pain after 15 repetitions, forcing the patient to stop because of lancinating pain. During the 6-minute walking distance test, the patient had to stop after 67 m and reported being unable to continue. Then, the ankle-brachial pressure index (ABI) [1,24] was taken before and after low-intensity cycling: the familiar leg pain was reproduced after 30 s of cycling, leading to stopping the test. However, the ABI was measured, showing a result of 0 on the affected side, as the systolic blood pressure was not detectable at the ankle.

### 2.3. Analysis and Clinical Action

The physical examination was suggestive of a severe peripheral vascular flow limitation and in need of an urgent referral. Although the patient was referred with a diagnosis of lower-back pain with sciatica and was previously visited by a vascular surgeon, who excluded the need for revascularization surgery after a Doppler ultrasound, many cues from the history prompted the suspicion of a potential serious vascular pathology. The patient’s reported risk factors (i.e., lifestyle, diet, comorbidities such as hyperglycemia and high cholesterol levels, previous cardiovascular comorbidities such as a popliteal artery thrombosis and an abdominal aortic aneurysm), the predictive skin changes, and the symptoms’ behavior, quality, and persistence were also clues. The subsequent vascular examination confirmed that the patient’s symptoms were likely due to a peripheral vascular disease (potentially femoral obliterative arteriopathy), allowing the physiotherapist to generate a clearer report for a medical physician [25]. With a high index of suspicion for a patient outside the scope of care, and with the goal of enhancing care continuity, the decision was made for a referral to a vascular surgeon. To make a “high-value” referral, the patient was informed about the clinical findings, and a report was prepared for the vascular surgeon.

### 2.4. Follow-Up

**Figure 3:**
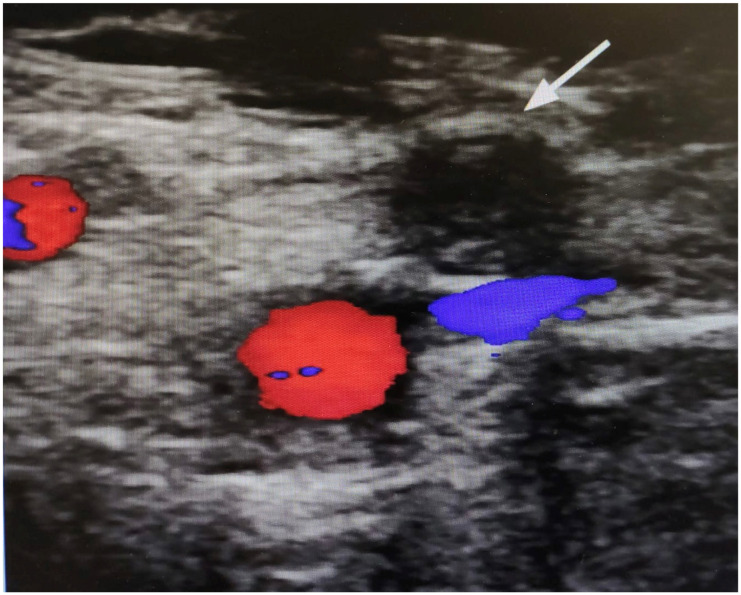
Doppler ultrasound image of the right thigh. The arrow highlights a complete obstruction of the superficial femoral artery. The absence of colors (red/blue) denotes a lack of flow.

The vascular surgeon observed slight pallor and hypothermia of the right foot, leading to the suspicion of an ischemic event of the right lower limb. Moreover, a Doppler ultrasound showed complete femoral artery obstruction (Figure 3). As the patient refused an additional surgery—mainly because of lack of confidence about being hospitalized due to the COVID-19 emergency—an additional therapy with phosphodiesterase type 3 inhibitors (one capsule in the morning and one in the evening) and possible infusion of iloprost was prescribed [26,27,28,29].

## 3. Discussion

This case report highlights the importance of screening for PAD in patients with signs and symptoms that raise suspicion of vascular pathology, even if a recent medical consultation has ruled out a vascular origin of the patient’s complaint. Notably, the final clinical presentation raises the notion that the vascular system must be considered as a whole, and a complete vascular examination must be performed to collect better anatomical clues to the potential pathology and the patient’s clinical presentation [1]. In addition, the location of the symptoms or signs (i.e., knee) must be considered in a systemic context to avoid any lack of recognition (i.e., femoral obliteration instead of a popliteal artery aneurysm), which could potentially have severe consequences [21].

Individuals with symptoms and risk factors that raise the suspicion of PAD should undergo a full neurological and cardiovascular examination driven by a sound clinical reasoning [1,14,18,19,20]. The neurological examination must include motor and sensory testing, because impairments in both of these domains may be indicative of further examination or the need for prompt intervention [7,30]. At the same time, the cardiovascular evaluation should comprise measurements of vital signs, blood pressure—on both upper extremities—and palpation/provocation of the brachial, radial, femoral, popliteal, dorsalis pedis, and posterior tibial arteries [10]. Clinical practice guidelines suggest that calculation of the resting ABI should also be included within the vascular examination, due to its high degree of sensitivity and specificity for PAD [1,10]. Finally, due to the impairments to the skin and tails frequently produced by PAD, the examination should include an inspection of the skin, tails, and limb temperatures [1].

For a comprehensive overview, we invite readers to refer to the step-by-step guide by Feller et al. [1] on objectively assessing a patient at risk of PAD. As illustrated in this case report, an early diagnosis of PAD is essential for managing and understanding the symptoms for both the clinician and the patient. The physiotherapist’s failure to identify PAD has the potential to delay a vascular consultation and, therefore, a proper medical management, exposing the patient to an increased risk of complications (e.g., stroke, amputation).

All patients with PAD, even if asymptomatic, should be referred to primary care, mainly for addressing modifiable risk factors and, eventually, pharmacological management [21]. The initial management should also comprise an exercise regimen for symptom reduction [10,31]. In more severe cases (e.g., claudication that affects the quality of life or the presence of critical limb ischemia), a referral to a vascular specialist consultation is suggested for further investigation with diagnostic imaging—such as duplex ultrasound and computed tomography angiography [32]. These examinations are needed to confirm PAD or any alternative vascular diagnosis (e.g., venous claudication, venous ulcer, distal small-arterial occlusion, autoimmune injury, and malignancy), which will determine the appropriate management pathway [10]. For those patients with chronic limb-threatening ischemia or disabling symptoms that are unresponsive to conservative management, percutaneous or surgical intervention is usually indicated [10].

## 4. Conclusions

Physiotherapists should be able to screen for PAD in people presenting with pain in the lower extremities. Failure to correctly screen for PAD could put the patient at risk of severe disability and possible permanent sequelae. This article presents a single episode of care and may represent an outlier in clinical practice, necessitating caution regarding the generalizability of our findings. However, this case report outlines the relevant concepts relating to the pathophysiology, screening, and differential diagnosis of PAD, and it further describes the relevant findings from the history and physical examination from the physiotherapist’s perspective in a patient with an unusual symptom presentation. Although the patient was referred by a physician with a diagnosis of LSR, our case also highlights the pivotal role of skilled physiotherapists in both primary and secondary care. Furthermore, physiotherapists could play a fundamental role in risk factor identification, early diagnosis, and subsequent appropriate management of cardiovascular disease.
